# Creatinine- and Cystatin C-Based Incidence of Chronic Kidney Disease and Acute Kidney Disease in AKI Survivors

**DOI:** 10.1155/2018/7698090

**Published:** 2018-09-27

**Authors:** Claire Rimes-Stigare, Bo Ravn, Akil Awad, Klara Torlén, Claes-Roland Martling, Matteo Bottai, Johan Mårtensson, Max Bell

**Affiliations:** ^1^Section of Anaesthesia and Intensive Care Medicine, Department of Physiology and Pharmacology, Karolinska Institute, Stockholm, Sweden; ^2^Perioperative Medicine and Intensive Care, Karolinska University Hospital, Stockholm, Sweden; ^3^Department of Cardiology, Södersjukhuset, Stockholm, Sweden; ^4^Department of Clinical Science and Education, Södersjukhuset, Karolinska Institute, Stockholm, Sweden; ^5^Division of Biostatistics, Institute of Environmental Medicine, Karolinska Institute, Stockholm, Sweden

## Abstract

**Background:**

Renal dysfunction after acute kidney injury (AKI) is common, potentially modifiable, but poorly understood. Acute kidney disease (AKD) describes renal dysfunction 7 to 90 days after AKI and is determined by percentage change in creatinine from baseline. Chronic kidney disease (CKD) is defined as the estimated glomerular filtration rate (eGFR) less than 60 ml/min/1.73 m^2^ persisting for more than 90 days^.^ We compared CKD incidence using both creatinine- and cystatin C-based GFR with AKD incidence at 90 days in AKI survivors.

**Methods:**

A prospective cohort study was conducted in a Swedish intensive care unit (ICU) between 2008 and 2010. We included AKI patients alive at 90 days. We excluded patients <18 and >100 years, death before follow-up, CKD prior to admission, and follow-up before 60 days or beyond 270 days. Creatinine and cystatin C were measured at 90 days and converted to eGFR (mL/min/1.73 m^2^).

**Results:**

We included 274 patients. At 90-day follow-up, the median creatinine eGFR (MDRD) was 81.6 (IQR 58.6–106.8) and median cystatin C eGFR was 51.5 (IQR 35.8–70.7). The incidence of CKD (eGFR < 60) was 25.8% based on creatinine but 63.7% using cystatin C estimates. AKD was present in 47 patients (18.9%). Age, discharge cystatin C, creatinine at discharge, and female gender predicted creatinine-defined CKD at follow-up. Age, discharge cystatin C, CRRT on ICU, and diabetes were associated with cystatin C-based CKD.

**Conclusions:**

In AKI survivors followed up at 3 months, CKD criteria were met in a quarter of patients using creatinine and in two-thirds using cystatin C eGFR. Less than one-fifth of patients fulfilled AKD criteria. The application of AKD criteria may underestimate renal dysfunction in AKI survivors.

## 1. Introduction

Acute kidney injury (AKI) in the critically ill is associated with an increased risk of chronic kidney disease (CKD), end-stage renal disease (ESRD), and elevated long-term mortality [1–4]. Follow-up of renal function after ICU is rare in Sweden as in many other countries; therefore, the true incidence of renal dysfunction after ICU is unknown and is almost certainly underestimated in registry studies.

Tentative evidence suggests that nephrological follow-up could improve mortality and possibly renal recovery [5, 6]. In a meta-analysis from 2012, Coca et al. demonstrated a tendency for a longer follow-up time to be associated with a reduction in CKD [3]. Intervention in the months after AKI could impede maladaptive renal repair which results in fibrosis and CKD. Attention is centred on establishing guidelines for viable follow-up programs [7, 8]. ADQI has focused research on the AKI recovery period and has given a precise definition to the previously proposed condition acute kidney disease (AKD). This is intended to allow greater consensus within the field and is defined as renal dysfunction persisting 7–90 days after AKI exposure. AKD is diagnosed by a minimum increase in creatinine of 1.5 times baseline and categorised into 5 groups [7]. Chronic kidney disease according to the KDIGO 2012 definition is an impairment of renal function or structure persisting for more than 90 days and corresponding to an estimated glomerular filtration rate (GFR) under 60 ml/min/1.73 m^2^ (link to KDIGO guidelines http://www.kdigo.org/clinical_practice_guidelines/pdf/KDIGO%20AKI%20Guideline.pdf). The use of creatinine as an endogenous marker of GFR is confounded in ICU patients due to fluid overload and importantly sarcopenia [9, 10]. How long the catabolic state continues after discharge and whether creatinine can be used in the recovery period are unclear. Cystatin C may be a superior marker in critical illness because it is not affected by muscle mass [11]. It is uncertain whether creatinine and cystatin C give similar estimates of the glomerular filtration rate (eGFR) in the months following discharge.

Surveillance of all post-AKI patients would be costly and impractical; we must therefore identify those patients most at risk of subsequent renal dysfunction and establish how to best determine renal function during the recovery period.

We conducted a follow-up study of AKI survivors three months after discharge. We aimed to identify the incidence of CKD according to both creatinine and cystatin C. Secondarily, we applied the newly defined AKD criteria and compared incidence of CKD with incidence of AKD at the transition period between the conditions. We also aimed to identify factors predictive of renal dysfunction at three months according to both endogenous biomarkers.

## 2. Methods

This is a prospective cohort study of patients with AKI admitted to our mixed ICU in Stockholm between September 2008 and May 2011. Ethical approval was granted by Stockholm Regional Ethics Committee, and the study was performed in accordance with the ethical standards laid down in the 1964 Declaration of Helsinki and its amendments.

AKI was defined according to RIFLE (current practice at recruitment time) using creatinine and urine output [12]. Patients were screened at discharge; those fulfilling RIFLE criteria at any point during ICU stay were eligible. We used convenience sampling to recruit AKI patients to the study. Recruitment occurred when research staff were in post. Patients discharged when research staff were not working or who were transferred to other hospitals were not recruited. Baseline creatinine was obtained from a review of laboratory and hospital admission data up to 3 months prior to ICU admission, and the lowest value was used as baseline. Where baseline was absent, creatinine was estimated using the modified diet in renal disease (MDRD) formula (expected GFR of 75 mL/min/1.73 m^2^) as recommended in KDIGO guidelines (http://www.kdigo.org/clinical_practice_guidelines/pdf/KDIGO%20AKI%20Guideline.pdf). Patients with absent baseline and no CKD diagnosis recorded prior to admission were presumed not to have preexisting CKD.

Adults with AKI during admission and alive at discharge were included. We excluded patients aged under 18 years and over 100 years, those who died before 3-month follow-up, and those with preexisting CKD. Only first admissions were analysed. Follow-ups occurring less than 60 days and greater than 270 days from admission were excluded. Recruited patients were referred either to nephrology or ICU clinics at 3 months when serum creatinine and cystatin C were measured.

We extracted information on all ICU admissions during the study period from our unit's data system, enabling us to identify non-AKI and nonrecruited AKI patients. This complete database was cross-matched with the Swedish death registry and Swedish renal register to obtain dates of death and ESRD diagnoses; thereafter, data were anonymised.

## 3. Statistical Methods

We report continuous data as medians with interquartile ranges (IQRs). Categorical data are expressed as counts and percentages. Mann–Whitney's test was used to compare distributions of continuous variables, the sign test tested equality of matched pairs, and Fisher's test compared means of binary variables. A two-sided *P* value <0.05 was considered statistically significant. Analysis was performed using Stata version 12 (StataCorp LP, College Station, TX, USA).

Values of creatinine were transformed to eGFR using the MDRD and Lund-Malmö (L-M) and CKD-EPI formulae. We used CKD-EPI (CKD-EPI-cy) to derive eGFR from cystatin C. A composite creatinine and cystatin C eGFR was calculated using the CKD-EPI (CKD-EPI-Cr-cy) combined formula (S1 GFR-estimating formulae). Patients were subsequently classified as having CKD at 3 months according to KDOQI stages of CKD; please note that urinalysis was not performed; therefore, categories 1 and 2 are denoted as GFR >90 and 60–90 ml/min/1.73 m^2^, respectively [13]. We classified patients (without prior CKD) as having AKD if their follow-up creatinine was >1.5 times their baseline creatinine.

### 3.1. Modelling

We considered death as a censoring event, without which we could have observed patients' biomarker values at 3 months. We created a model using Cox regression weighted for the inverse probability of dying after discharge and before 3 months and adjusted for covariates found to be independently associated with death before follow-up. The following variables were included in the final model: age, sex, and maximum RIFLE level. This model was used in all regression analyses.

In all modelling, potential confounders were considered on the basis of prior knowledge of AKI and predictors of mortality using variables in Table 1, including length of stay and creatinine/cystatin C ratio (both surrogate markers of sarcopenia). Univariate analysis of each covariate was performed, and variables with a *P* value less than 0.1 were selected as candidates for the multivariate analysis. Multivariate analyses were conducted using the stepwise backward elimination technique using a significance level of 10%, and we tested for collinearity.

Logistic regression was used to identify covariates which affected the risk of the binary outcome CKD (GFR <60 mL/min/1.73 m^2^) according to (i) creatinine and (ii) cystatin C. Odds ratios are presented. The models were assessed using Somers' *d* as well as Bayesian and Akaike information criteria (BIC and AIC).

Survival probabilities were calculated using the Kaplan–Meier method, and differences between groups were tested using the log-rank test.

## 4. Results

**Figure 1:**
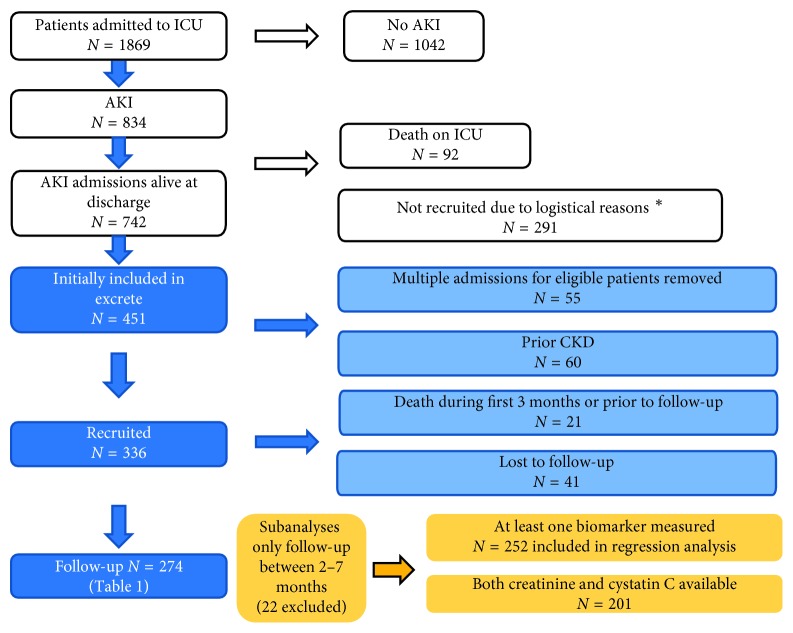
Flow chart showing selection and exclusion of patients in the follow-up cohort. White boxes: entire ICU cohort, information derived from cross-matching with the ICU database and death register; blue boxes: data from the initial study database; yellow boxes: details of groups included in subanalyses. ^*∗*^Patients discharged when research staff were not working or who were transferred to other hospitals were not recruited.

During the study time, 1869 ICU patients were admitted and 41.4% fulfilled RIFLE criteria for AKI. ICU mortality was 11.1% among AKI patients. Of those alive at discharge, 336 entered the study, 41 lost to follow-up, and 21 died before follow-up (Figure 1). Reasons for loss to follow-up included moving outside of the region, diagnosis of dementia, social problems, and feeling too unwell to attend. Table 1 presents baseline characteristics of the remaining recruited patients. Details of the entire ICU cohort are given in Table S2. The median follow-up was 101.5 days (IQR 89.5–126). Admission reasons for the recruited patients are displayed in Figure S3.

Recruited AKI patients had a median age of 64 years, and 41.6% were female; the median SAPS-2 score was 48.5 (Table 1). The studied groups were similar in age, gender distribution, and SAPS-2 score to nonrecruited AKI patients (included patients who died before follow-up) (Table S2). Recruited patients had a longer length of stay (LOS), a lower proportion received invasive ventilation, and the median daily diuresis was higher than that in the nonrecruited group.

Patients without AKI were younger, had shorter LOS, and lower SAPS-2 scores than AKI patients. Non-AKI patients had a lower frequency of invasive ventilation and lower creatinine, urea, and cystatin C throughout their ICU stay (Table S2).

Baseline creatinine was measured in 56.9% of patients, median was 64 *µ*mol/l (IQR 50.5–76), and median estimated baseline creatinine was 88 *µ*mol/l (71–97) (Table 1). The median follow-up creatinine was 76 *µ*mol/l (IQR 59–96) (Table S4). Cystatin C was available in 211 patients with a median of 1.33 mg/l (IQR 1.09–1.73).

GFR estimates for patients where both creatinine and cystatin C were assessed are presented in Table 2. The Lund-Malmö formula gave the lowest median GFR estimate of the creatinine-based formulae (74.6 compared with 81.6 mL/min/1.73 m^2^ for MDRD; *P* ≤ 0.001), which is nearer to the cystatin C median GFR (51.4 mL/min/1.73 m^2.^). Estimates obtained using the combined cystatin C and creatinine formula (64.5 mL/min/1.73 m^2^) lie in between estimates from individual markers.

## 5. Patients Meeting Chronic Kidney Disease Criteria

**Figure 2:**
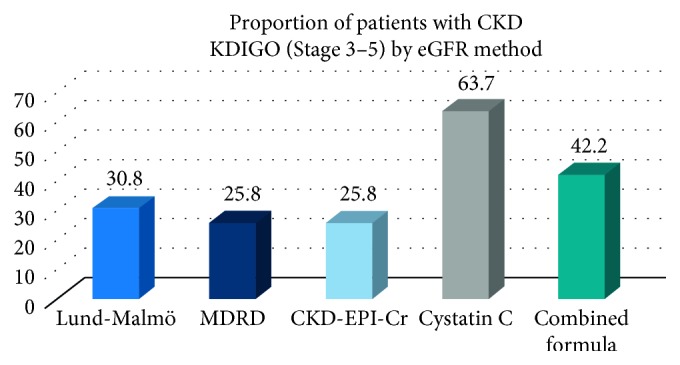
Categorisation of patients by CKD group (stage 3–5) at follow-up according to the method of GFR estimation, in 201 patients where both variables were measured.

63.7% of patients fulfilled the criteria for KDIGO CKD stage 3 or greater at first follow-up using cystatin C-based estimates, whereas when creatinine-based formulae were applied 30.8% fulfilled CKD criteria using L-M and 25.8% according to both MDRD and CKD-EPI-based estimates. (Figure 2 and Table S5). The combined formula identified 42.2% of patients as having CKD.

## 6. Acute Kidney Disease Diagnosis

In 252 patients in whom follow-up creatinine was obtained between 2 and 7 months, 47 (18.7%) fulfilled AKD criteria. Of 201 patients with both biomarkers available (the same group as eGFR analysis), 38 (18.9%) met AKD criteria (Table 3).

Incidence of AKD at ICU discharge could not be described because the groups' median length of stay (6 days) was less than the minimum time when AKD may be diagnosed (7 days after AKI insult). However, analysis showed that, on the day of discharge, 204 patients (60.7%) fulfilled AKD criteria.

Multivariate logistic regression estimated the risk of CKD (eGFR <60 mL/min/1.73 m^2^) according to creatinine (MDRD) and cystatin C. Two models for creatinine-based CKD are shown in Table 4. Both models include age and female gender, and model-1 also includes cystatin C at discharge; this performed somewhat better when assessed using Somers' *d*, AIC, and BIC than model-2 which used discharge creatinine. Covariates associated with 3-month cystatin C-eGFR (Table 5) were age, cystatin C at discharge, and diabetes. Univariate sensitivity analysis showed that the likelihood of diagnosis with CKD according to cystatin C and the likelihood of AKD increased significantly if known baseline creatinine was used rather than estimated baseline, and this effect disappeared in multivariate analysis.

One-year mortality was 18.7% in non-AKI patients and 28.2% in all AKI patients. Two-year mortality was significantly higher for patients with creatinine-based CKD (23.1%) than no CKD (8.1%) (*P*=0.022). Mortality did not significantly differ between patients classified as having CKD according to creatinine- or cystatin C-based CKD (13.2%) (*P*=0.162); neither did mortality vary according to AKD status. The characteristics of patients classified according to CKD status at 3 months are presented in Table 6. Only 2 patients had CKD based on creatinine but not cystatin C; their data are not presented in the table.

## 7. Recording of Chronic Kidney Disease and End-Stage Renal Disease Diagnoses in National Registers

Of the 336 patients initially recruited to the study without prior CKD, 14 were subsequently registered by nephrologists in the Swedish National Patients Register as having CKD (4.1%) and 4 (1.2%) were recorded in the Swedish Renal Register as having developed ESRD. In the non-AKI group, 1 patient (0.1%) was subsequently registered as ESRD.

## 8. Discussion

### 8.1. Key Findings

In ICU patients studied three months after AKI, the incidence of CKD using creatinine-based formulae varied between 25.8 and 30.3% and was 63.7% when cystatin C-based eGFR was applied; concurrently, 18.9% of patients fulfilled AKD criteria.

### 8.2. Comparison to Other Studies

CKD incidence of at least 25% in this follow-up study may be compared to incidence in registry studies such as our own previous national study where incidence was recorded at 6% (albeit at 1 year) [14]. Even given that some renal recovery may occur over the first year, the discrepancy is large and may be due to underdiagnosis in patients not clinically followed. Indeed, only 4.1% of the patients in this study received an official diagnosis of CKD in the national registers.

Macedo studied 84 AKI survivors and found renal recovery occurring up to one and a half years after ICU, and 36% had creatinine-estimated GFR <60 mL/min/1.73 m^2^ at 18 months [15]. Ponce discovered 43.3% of 500 Brazilian AKI grade 3 patients with CKD at 36 months [16]. Similarly, an Islandic register study of over 25,000 patients with hospital AKI reported renal recovery (return to <1.5 times the baseline creatinine) ranged from 88% to 44% in AKI grades 1–3, respectively [17]. A Scottish population study of over half a million people found that, after AKI, 68% of patients returned to a “threshold level” (creatinine <150 *µ*mol/l for men and < 130 *µ*mol/L for women) [18].

CKD incidence was far higher when GFR was estimated using cystatin C than with creatinine, and this finding is novel. At the steady state, these biomarkers give similar estimates of GFR, acceptably close to measured values [19, 20]. Differences seen in our study could be due to a number of factors, including sarcopenia occurring during ICU admission and continuing to affect creatinine values in the recovery period. We found cystatin C at discharge better predicted presence of AKD and creatinine-based CKD at follow-up than discharge creatinine did, and this supports previous research suggesting that discharge creatinine is confounded as a renal marker. However, creatinine/cystatin C ratio unexpectedly decreased from discharge to follow-up, and length of stay (LOS) (both proxies for ICU muscle mass loss) was not independently associated with follow-up creatinine or cystatin C. Hence, this study does not demonstrate that the effect of sarcopenia extends to affect 3-month creatinine.

The explanation may lie in the difference in ability of cystatin C- and creatinine-based equations to estimate measured GFR in certain populations. This has been seen in elderly patients. Two community-based studies of the elderly found CKD-EPI-Cr-cy to have the greatest accuracy [21], whilst creatinine-based equations particularly lacked accuracy at GFR under 45 [22]. Alternatively, other confounders may explain the discrepancy between eGFR methods; hypothetically, factors affecting cystatin C such as corticosteroid use or thyroid dysfunction during recovery could be important.

It is also conceivable that cystatin C may be reflecting something other than GFR. It may reflect tubular or extra renal function. Shlipak et al. demonstrated in an elderly population that elevated cystatin C in patients with creatinine-based eGFR greater than 60 was predictive of subsequent development (4 years later) of CKD, cardiovascular disease, and death [23]. The explanation for this is unclear. Cystatin C is freely filtered, reabsorbed, and fully metabolised in the tubule. Could metabolism be impaired in renal failure, or does cystatin C reflect a nonrenal signal?

The incidence of AKD when applied to 3-month creatinine values was lower (18.9%) than the incidence of creatinine-based CKD (25–30%). The concept of AKD in providing a consensus definition of post-AKI renal dysfunction is commendable, and the definition itself may be problematic for a number of reasons. Firstly, the definition relies on a correct baseline creatinine being available which is often not. It may be more relevant to know whether the patient has renal dysfunction (CKD) in the recovery period than to relate their current function to a real or assumed baseline; otherwise, both AKI and AKD risk are being misclassified. Secondly, AKD diagnosis is broadly based on AKI diagnostic criteria, which are well validated and accurate in identifying AKI and predicting mortality but may not necessarily be good at predicting renal dysfunction or recovery after AKI. Additionally, basing AKD on creatinine may mean classification is affected by the problems (discussed above) inherent with creatinine use after ICU stay such as sarcopenia, particularly if applied in the first week after discharge.

Diagnosis of CKD according to creatinine-based eGFR was associated with significantly higher mortality than no CKD at 3 months. However, the study was underpowered to detect mortality differences, and a larger investigation with longer follow up is required to definitively determine mortality differences depending on the biomarker used to classify CKD.

### 8.3. Study Strengths

The prospective design meant that patients with all grades of AKI were studied and GFR measurements occurred at a predetermined time, thus largely avoiding the selection bias of creatinine been measured due to clinical indication. Although there was some loss to follow-up of renal function estimation, we have complete follow-up of mortality and ESRD incidence (up to 5 years) in all ICU patients due to access to the highly reliable Swedish health registries [24]. The study included relatively a large number of patients compared to other clinical follow-ups, and highly detailed information regarding comorbidities, in-hospital parameters, and treatments were available.

### 8.4. Study Limitations

This study was conducted by convenience sampling. We were unable to recruit all AKI patients alive at discharge due to limited availability of research staff. We recruited 61% of AKI patients who were alive at discharge (prior to exclusions). Loss to follow-up is present and was due in part to logistical difficulties and in 12.8% of cases was patient related. Urinalysis was unavailable and would have allowed full CKD classification according to KDGIO staging. The study could have been strengthened by the presence of a non-AKI control group; however, limited resources prevented this. At study inception, MDRD was the method chosen to estimate baseline creatinine when baseline was absent. MDRD was also used to subsequently RIFLE classify patients at recruitment. Other equations have since been shown to be more accurate in estimating GFR. We therefore present other creatinine-based equations including the Lund-Malmo equation (based on a Swedish population). If MDRD baseline estimates differed from true values, AKI may have been misclassified. Sensitivity analysis found that use of known baseline creatinine rather than estimated was not independently associated with the likelihood of subsequent diagnosis with CKD or AKD.

### 8.5. Significance

This study is unique in using cystatin C to measure renal function in AKI survivors and to compare incidence with creatinine-based CKD. Furthermore, in presenting and comparing AKD incidence and CKD incidence at the same time point after AKI, it is novel. The discrepancy observed between creatinine and cystatin C GFR estimates is important because creatinine is the established method for eGFR and is recommended by ADQI to define AKD [7]. Patients at risk of chronic renal dysfunction may be missed if creatinine is the sole renal function marker used to assess eGFR in AKI survivors, particularly if AKD criteria alone are applied. Refinement and validation of AKD criteria may be necessary. Further research comparing creatinine- and cystatin C-based GFR estimates with the gold standard GFR measurement after AKI is required.

## 9. Conclusion

Three months after AKI, significant renal impairment persisted in AKI survivors being at least 25% according to creatinine-based CKD and 67% when classified using cystatin C-estimated GFR. AKD criteria identified fewer patients with renal dysfunction than CKD criteria did. Use of creatinine alone at follow-up may lead to underestimation of renal dysfunction in AKI survivors, and AKD criteria may require revision.

## Figures and Tables

**Table 1:** Baseline characteristics of all recruited AKI patients.

Baseline characteristics of all recruited AKI patients (274)	Median values and IQR unless otherwise stated
Median age (years) (IQR)	64 (53–72)
Sex (female), *N* (%)	114 (41.6)
Length of stay (days) (IQR)	6 (3–12)
SAPS-2 score (IQR)	48.5 (38–64)
Invasive ventilation, *N* (%)	109 (40)
Dialysis on ICU, *N* (%)	66 (24)
Maximum urea (mmol/l) (IQR)	15.7 (9–25.2)
Baseline creatinine	
** ** *N* measured (%)	156 (56.9)
** **Measured (*µ*mol/l) (IQR)	64 (50.5–76)
** **Estimated^a^ (*µ*mol/l) (IQR)	88 (71–97)
** ** *N *= 146	
Admission creatinine (*µ*mol/l) (IQR)	135 (104–213)
Maximum creatinine (*µ*mol/l) (IQR)	169.5 (122–263)
Last ICU creatinine (*µ*mol/l) (IQR)	107 (72–149.5)
Admission cystatin C (mg/l) (IQR)	1.58 (1.1–2.35)
Maximum cystatin C (mg/l) (IQR)	2.14 (1.44–3.04)
Last ICU cystatin C (mg/l) (IQR)	1.65 (1.23–2.21)
Discharge creatinine/cystatin C ratio (IQR)	7.1 (5.2–9.2)
COPD	53 (14.5)
Diabetes mellitus I and II	54 (19.7)
Cardiovascular disease	90 (33.0)
Hypertension	120 (44.0)
Liver failure	99 (36.0)
Haematological malignancy	19 (6.9)
Other malignancies	84 (30.6)
Heart failure	38 (13.8)

MDRD = modified diet in renal disease formula. ^a^Creatinine was estimated using the Modified Diet in Renal Disease (MDRD) formula using an expecting GFR of 75 ml/kg/min/1.73 m^2^.

**Table 2:** Median eGFR in 201 patients where both variables were measured with follow-up between 2 and 7 months.

GFR estimates at 3-month follow-up, according to creatinine- and cystatin C-based equations					
*N*=201 (mL/min/1.73 m^2^)	Median	IQR	Minimum	Maximum	*P* compared to L-M estimate
Lund-Malmö	74.6	55.9–94.3	18.5	132.2	Reference
MDRD	81.6	58.6–106.8	7.0	225.2	<0.001
CKD-EPI-Cr	86.0	59.6–101.4	6.6	139.6	<0.001
CKD-EPI-cy	51.4	35.8–69.9	9.1	138.3	<0.001
CKD-EPI-Cr-cy	64.5	46.7–83.5	7.28	137.5	<0.001

L-M = Lund-Malmö formula. MDRD = modified diet in renal disease formula.

**Table 3:** Categorisation of the cohort according to the AKD group, in 201 patients where creatinine and cystatin C were both measured with follow-up between 2 and 7 months.

Acute kidney disease grade	*N*	%
0	82	40.8
0 B-C	81	40.3
1	26	12.9
2	8	3.98
3	4	1.99
AKD grade 1–3	38	18.9

**Table 4:** Logistic regression model presenting odds ratios for estimates of CKD (GFR under 60 mL/min/1.73 m^2^) according to creatinine at follow-up analysis weighted for the risk of death before follow-up. Follow-up was between 2 and 7 months. Probability of CKD was according to MDRD creatinine-based eGFR < 60 ml/min/1.73 m^2^ at follow-up.

Covariate	Model 1		Model 2	
	Odds ratio (95% CI)	*P*	Odds ratio (95% CI)	*P*
Discharge cystatin C (mg/l)				
** **0–2	1.0 (ref)			
** **2-3	2.3 (1.1–4.8)	<0.031^*∗*^		
** **>3	4.6 (1.4–15.2)	<0.013^*∗*^		

Discharge creatinine (*µ*mol/l)				
** **<100			1.0 (ref)	
** **100–200			2.3 (1.1–4.7)	0.025^*∗*^
** **200–300			2.9 (1.0–8.2)	0.050
** **>300			4.7 (0.5–44.4)	0.179

Age (years) (25 centile distribution)				
** **<52	1.0 (ref)		1.0 (ref)	
** **52–64	1.9 (0.5–8.6)	0.363	2.2 (0.5–9.9)	0.293
** **64–72	8.0 (2.1–30.9)	0.003^*∗*^	8.8 (2.1–34.8)	0.002^*∗*^
** **>72	11.8 (2.9–30.9)	<0.001^*∗*^	14.1 (3.6–55.1)	<0.001^*∗*^

Gender				
** **Male	1.0 (ref)		1.0 (ref)	
** **Female	3.0 (1.5–6.1)	0.002^*∗*^	3.4 (1.7–6.9)	0.001^*∗*^

^*∗*^ *P* = 0.05 significance level.

**Table 5:** Logistic regression model presenting odds ratios for estimates of CKD (GFR under 60 mL/min/1.73 m^2^) according to cystatin C at follow-up analysis weighted for the risk of death before follow-up. Follow-up was between 2 and 7 months. The probability of CKD was according to CKD-EPI cystatin C-based eGFR <60 ml/min/1.73 m^2^ at follow-up.

Covariate	Odds ratio (95% CI)	*P*
Discharge cystatin C (mg/l)		
** **0–2	1.0 (ref)	
** **2-3	2.2 (0.9–5.02)	0.005^*∗*^
** **>3	3.8 (1.1–13.0)	0.061^*∗*^

Age (years) (25 centile distribution)		
** **<52	1.0 (ref)	
** **52–64	6.4 (1.9–21.2)	0.002^*∗*^
** **64–72	17.6 (5.3–58.1)	<0.001^*∗*^
** **>72	78.2 (18.6–329)	<0.001^*∗*^

CRRT in ICU		
** **No	1.0	
** **Yes	3.32 (1.3–8.6)	0.013

Comorbidity		
** **No diabetes	1.0 (ref)	
** **Diabetes I and II	2.7 (1.0–7.4)	0.057

^*∗*^ *P* less than 0.05 significance level.

**Table 6:** Patient characteristics according to CKD classification at three months.

Variable	CKD status at 3 months (*N *= 199)^*∗*^				
	No CKD (*N*=74)	CKD creatinine and cystatin C (*N*=52)	*P* compared to no CKD	CKD cystatin C only (*N*=83)	*P* compared to no CKD
Age	51.5 (35–62)	72.5 (66.5–81.5)	<0.001^*∗*^	68 (60–73)	<0.001^*∗*^
Gender (female)	31 (41.9%)	29 (55.8%)	0.149	30 (36.2%)	0.513
Hypertension	18 (24.2%)	34 (65.4%)	<0.001^*∗*^	46 (55.4%)	<0.001^*∗*^
Cardiovascular disease	14 (18.9%)	20 (38.5%)	0.024^*∗*^	33 (39.8%)	0.005^*∗*^
Diabetes	8 (10.8%)	18 (34.6%)	0.002^*∗*^	19 (22.9%)	0.057
COPD	6 (8.1%)	10 (19.3%)	0.101	15 (18.1%)	0.099
Heart failure	6 (8.1%)	9 (17.3%)	0.162	11 (13.4%)	0.316
Invasive ventilation	34 (46%)	21 (40.4%)	0.587	34 (41%)	0.627
CRRT	10 (13.5%)	14 (26.9%)	0.069	27 (32.5%)	0.008^*∗*^
LOS	5 (3–8)	5.5 (3–12)	0.783	8 (3–15)	0.189
Mortality at 2 years	8.1%	23.1%	0.022^*∗*^	13.2%	0.441

^*∗*^One hundred ninety-nine patients because 2 of 201 patients in whom both biomarkers were measured at follow-up 2–7 months had CKD based on creatinine but not cystatin C; their data are not presented here.

## Data Availability

The data used to support the findings of this study are included in anonymised form within the supplementary material file.

## Supplementary Materials

Supplementary Materials S1: GFR estimating formulae. Table S2: baseline characteristics of all ICU patients. Figure S3: admission reason for recruited patients. Table S4: creatinine and cystatin C values for patients who attended follow-up at any time. Table S5: CKD stage according to creatinine- and cystatin C-based equations. S6: study database (excel file).
