# Supplementary material for: Creatinine- and Cystatin C-Based Incidence of Chronic Kidney Disease and Acute Kidney Disease in AKI Survivors
Source: Crit Care Res Pract. 2018 Sep 27;2018:7698090. doi: 10.1155/2018/7698090 (PMC6180984; doi:10.1155/2018/7698090)
Supplement: Supplementary Materials — S1: GFR estimating formulae. Table S2: baseline characteristics of all ICU patients. Figure S3: admission reason for recruited patients. Table S4: creatinine and cystatin C values for patients who attended follow-up at any time. Table S5: CKD stage according to creatinine- and cystatin C-based equations. S6: study database (excel file). [file 7698090.f1.zip › 7698090.f1/mat.7698090.v2.docx]

**Supplementary materials**

**Contents:**

1. S1. GFR estimating formulae.
2. S2. Table: Baseline characteristics of all ICU patients
3. S3. Figure: Admission reason for recruited patients.
4. S4 table Creatinine and Cystatin C values for patients who attended followed-

up at any time

1. S5 Table. CKD stage according to creatinine and cystatin C based equations.
2. S6 Study database (excel)

**S1. GFR estimating formulae.**

**Revised Lund-Malmö equation**

eX – 0.0158×Age + 0.438×ln(Age)

Female   pCr < 150 μmol/L:   X= 2.50 + 0.0121 × (150 – pCr)

Female   pCr ≥ 150 μmol/L:   X= 2.50 – 0.926 × ln(pCr/150)

Male   pCr < 180 μmol/L:   X= 2.56 + 0.00968 × (180 – pCr)

Male   pCr ≥ 180 μmol/L:   X= 2.56 – 0.926 × ln(pCr/180)

**CKD-EPI Study equation for Caucasians**

Female   pCr  ≤  62 μmol/L:   44 × (pCr/62) − 0.329 × 0.993^Age^

Female   pCr > 62 μmol/L:  144 × (pCr/62) − 1.209 × 0.993^Age^

Male   pCr  ≤  80 μmol/L: 141 × (pCr/80) − 0.411 × 0.993 ^Age^

Male   pCr > 80 μmol/L:   141 × (pCr/80) − 1.209   × 0.993 ^Age^

**MDRD Study equation for caucasians**

175 × (pCr/88.4) − 1.154   × Age − 0.20 3   × 0.742 (if female)

Formulas above obtained from “*The revised Lund-Malmo GFR estimating equation outperforms MDRD and CKD-EPI across GFR, age and BMI intervals in a large Swedish population” Nyman, Grubb, Larsson, Björk. Clin Chem lab med 2013.* <https://doi.org/10.1515/cclm-2013-0741>

**CKD_EPI Cystatin C equation 2012**

eGFR =133 x min(S_cys_/0.8, 1)^-0.499^ x max (S_cys_/0.8, 1)^-1.328^ x 0.996^Age^ x0.932 [if female]

*Obtained from National Kidney Foundation website:* [*https://www.kidney.org/content/ckd-epi-cystatin-c-equation-2012*](https://www.kidney.org/content/ckd-epi-cystatin-c-equation-2012) Last accessed 2017-07-29

***CKD-EPI Cystatin C and Creatinine equation 2012***

eGFR =135 × min(S_Cr_/κ, 1)^α^ × max(S_Cr_/κ, 1)^-0.601^ × min(S_cys_/0.8, 1)^-0.375^ ×max(S_cys_/0.8, 1)^-0.711^ ×
0.995^Age^ ×0.969 [if female] ×1.08 [if black]

*Formula above obtained from National Kidney foundation website:* [*https://www.kidney.org/content/ckd-epi-creatinine-cystatin-equation-2012*](https://www.kidney.org/content/ckd-epi-creatinine-cystatin-equation-2012) Last accessed 2017-07-29

**Table S2.** Baseline characteristics of all ICU patients

| Baseline Characteristics  of all patients | All patients  N=1869 | No AKI  N=1042 | P  No-AKI vs All-AKI  (N=827) | AKI not recruited  N=491 | All initially  recruited AKI patients  N=336* | P  AKI not recruited vs AKI recruited. |
| --- | --- | --- | --- | --- | --- | --- |
| Mean age **(years) (SD)** | 55.6(19.4) | 51.2(19.9) | <0.001* | 61.2(17.9) | 60.8(16.3) | 0.427 |
| Sex, **% female (N)** | 38.2 (714) | 37.5 (391) | 0.498 | 38.5 (189) | 39.8 (134) | 0.688 |
| Median length of Stay **(days) (IQR)** | 3 (2-6) | 3 (2-4) | <0.001* | 4 (3-7) | 6 (3-12) | <0.001* |
| Mean SAPS 2 score  **(SD)** | 42.7 (19.8) | 35.9(17.3) | <0.001* | 51.0 (20.3) | 50.3(18.4) | 0.641 |
| Invasive ventilation, **% (N)** | 41.3 (771) | 37.6 (392) | <0.001* | 49.7 (244) | 40.2(135) | 0.007* |
| Dialysis on ICU, **% (N)** | 7.5(140) | 0 (0) | <0.001* | 12.0 (59) | 24.1 (81) | <0.001* |
| Mean lowest daily diuresis, **(ml), (SD)** | 1125 (984) | 1449(1024) | <0.001* | 553(619) | 990(883) | <0.001* |
| Mean maximum Urea **mmol/l (SD)** | 11.6 (17.1) | 6.1 (10.9) | <0.001* | 15.3 (16.5) | 19.5 (24.3) | <0.001* |
| Mean admission Creatinine**(umol/l) (S.D.)** | 127.3 (132) | 73.6 (22.3) | <0.001* | 183 (195.6) | 179(125.6) | <0.001* |
| Mean maximum Creatinine **(umol/l) (SD)** | 143 (141) | 77 (23.0) | <0.001* | 208 (202.5) | 212(131.7) | <0.001* |
| Mean last ICU Creatinine **(mmol/l) (S.D.)** | 106.5(96.3) | 67.9(21.3) | <0.001* | 156 (140.0) | 130.3(93.8) | 0.285 |
| Mean admission Cystatin C **(mg/l) (S.D.)** | 1.1 (1.04) | 0.63 (0.52) | <0.001* | 1.62 (1.36) | 1.8 (0.99) | 0.061 |
| Mean maximum Cystatin C **(mg/l) (S.D.)** | 1.71 (1.23) | 1.04 (0.45) | <0.001* | 2.31 (1.56) | 2.38 (1.13) | 0.003* |
| Mean last ICU Cystatin C **(mg/l) (S.D.)** | 1.17 (1.1) | 0.66 (0.56) | <0.001* | 1.80 (1.47) | 1.8 (0.93) | 0.199 |

***includes 41 patients who were lost to follow-up (non-attendance), 21 who died before follow-up and 274 who attended.**

**Fig S3. Reason for admission for patients or all initially recruited to the study.**

**Table S4.** Creatinine and Cystatin C values for patients who attended followed-up at any time

| Variable | N | Median | IQR |
| --- | --- | --- | --- |
| Baseline creatinine umol/l | 274 | 71 | 62-88 |
| Maximum ICU creatinine umol/l | 271 | 169 | 122-262 |
| Last ICU creatinine umol/l | 271 | 107 | 72-149 |
| Last ICU cystatin C mg/L | 274 | 1.65 | 1.23-2.2 |
| Discharge Creatinine/ cystatin ratio | 196 | 7.1 | (5.3-9.2) |
| 3-month follow-up | | | |
| Creatinine umol/l | 274 | 76 | 59-96 |
| Creatinine GFR (L-M) * | 274 | 76.5 | 57.2-96.1 |
| Cystatin C mg/l | 211 | 1.33 | 1.09-1.73 |
| Cystatin C GFR | 211 | 51.5 | 35.8-70.7 |
| Follow-up creatinine/ cystatin C ratio | 201 | 6.6 | (5.5-8-1) |

**Table S5** *KDOQI* CKD stage according to creatinine and cystatin C based equations, in 201 patients where both variables were measured.

| *KDOQI*  CKD stage | Estimated GFR formula | | | | |
| --- | --- | --- | --- | --- | --- |
|  | Creatinine Based formulae | | |  | |
|  | L-M^a^ | MDRD^b^ | CKD-EPI | CKD-EPI  Cystatin C | Combined CKD-EPI  Creatinine & Cystatin C |
|  | N (%) | | | | |
| GFR >90 * | 61 (30.4) | 80 (39.8) | 86 (42.8) | 28 (13.9) | 42 (20.9) |
| GFR 60-90* | 78 (38.8) | 69 (34.3) | 63 (31.3) | 45 (22.4) | 74 (36.8) |
| 3 | 55 (27.4) | 45 (22.4) | 44 (21.9) | 97 (48.3) | 71 (35.3) |
| 4 | 7 (3.5) | 6 (3.0) | 7 (3.48) | 24 (11.9) | 12 (6.0) |
| 5 | 0 (0) | 1 (0.5) | 1 (0.5) | 7 (3.5) | 2 (1.0) |
| CKD (Stages 3,4 5) | 62 (30.8) | 52 (25.8) | 52 (25.8) | 128 (63.7) | 85 (42.2) |

^a Lund-Malmö formula^

^b MDRD = Modified Diet in Renal Disease formula.^

^*no urine analysis available^
